# How to Obtain NNT from Cohen's d: Comparison of Two Methods

**DOI:** 10.1371/journal.pone.0019070

**Published:** 2011-04-27

**Authors:** Toshi A. Furukawa, Stefan Leucht

**Affiliations:** 1 Department of Health Promotion and Human Behavior (Cognitive-Behavioral Medicine), Kyoto University Graduate School of Medicine/School of Public Health, Kyoto, Japan; 2 Department of Psychiatry and Psychotherapy, Technische Universität München, Klinikum rechts der Isar, Munich, Germany; University of Modena and Reggio Emilia, Italy

## Abstract

**Background:**

In the literature we find many indices of size of treatment effect (effect size: ES). The preferred index of treatment effect in evidence-based medicine is the number needed to treat (NNT), while the most common one in the medical literature is Cohen's d when the outcome is continuous. There is confusion about how to convert Cohen's d into NNT.

**Methods:**

We conducted meta-analyses of individual patient data from 10 randomized controlled trials of second generation antipsychotics for schizophrenia (n = 4278) to produce Cohen's d and NNTs for various definitions of response, using cutoffs of 10% through 90% reduction on the symptom severity scale. These actual NNTs were compared with NNTs calculated from Cohen's d according to two proposed methods in the literature (Kraemer, et al., *Biological Psychiatry*, 2006; Furukawa, *Lancet*, 1999).

**Results:**

NNTs from Kraemer's method overlapped with the actual NNTs in 56%, while those based on Furukawa's method fell within the observed ranges of NNTs in 97% of the examined instances. For various definitions of response corresponding with 10% through 70% symptom reduction where we observed a non-small number of responders, the degree of agreement for the former method was at a chance level (ANOVA ICC of 0.12, p = 0.22) but that for the latter method was ANOVA ICC of 0.86 (95%CI: 0.55 to 0.95, p<0.01).

**Conclusions:**

Furukawa's method allows more accurate prediction of NNTs from Cohen's d. Kraemer's method gives a wrong impression that NNT is constant for a given d even when the event rate differs.

## Introduction

When a clinician and a patient jointly decide on a treatment, they need to know how much the treatment in question is better than an alternative treatment and in what respect. Effect size (ES) is an index, a single number preferably, that expresses this HOW MUCH.

Clinical decision-making is facilitated by consideration of the difference in risk of important beneficial (e.g. remission of an episode) or adverse (e.g. suicide) events or the reciprocal of this risk difference, the number needed to treat (NNT) [1], [2], [3]. The NNT is defined as the number of patients one would need to treat with the intervention in question in order to have one more success (or one less failure) than if treated in the control intervention. It is calculated by the following formula:
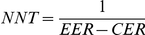
where EER is the experimental event rate and CER is the control event rate. For example, if the response rate in the acute phase treatment of a major depressive episode is 60% in the active drug arm (EER) and 30% in the placebo arm (CER), the NNT will be calculated as 1/(0.6−0.3) = 3.3. In order to simplify the argument, here and in the following, we assume that an intervention aims at increasing the event rate, so that EER is greater than CER. When an intervention is a preventative one, we need to exchange EER and CER appropriately.

When the outcome is continuous, however, the most common summary ES index in the medical literature is Cohen's d [4]. Clinicians and patients may find it challenging to understand the magnitude of effect in terms of Cohen's d, and so it is deirable to express results as a risk difference or NNT but conversion from Cohen's d to NNT is not self-evident. One of the authors has once proposed a conversion table from Cohen's d to NNT, under the assumption of normal distributions and equal variances in the intervention and control groups [5]. In this approach NNT is dependent on the threshold to define response on the continuous scale. Using the CER that corresponds with this threshold,
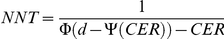
where Φ is the cumulative distribution function of the standard normal distribution and Ψ is its inverse. This formula shows that, given a certain Cohen's d, NNT will differ according to the response threshold you expect and the CER associated with that threshold.

Recently Kraemer and Kupfer [6] reviewed the commonly used ES indices and, based on the principles of statistical significance and power, recommended area under the receiver operating characteristics (AUC) comparing treatment and control responses, success rate difference (SRD), and number needed to treat (NNT). AUC is defined as the probability that a patient in the treatment has an outcome preferable to one in the control, and SRD as the difference between the probability that a patient in the treatment has an outcome preferable to one in the control and the probability that a patient in the control has an outcome preferable to one in the treatment. Thus,
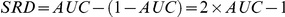
They further demonstrated that, when Cohen's d is appropriate (normal distributions, equal variances), it can be converted into AUC by the formula:
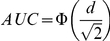
Therefore,
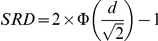
NNT is then calculated as:
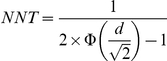
This NNT can therefore be interpreted as the number of patients one would need to treat with the intervention in order to have one more patient to have an outcome better than a randomly selected one in the control group than if the same number had been given the control intervention. This definition is clinically abstract and beyond comprehension of even well-informed clinicians and patients. However, it has been used in several recent important meta-analyses to quantify the obtained effect size [7], [8], [9]. As can be easily seen from the formula, this NNT is constant, given a certain Cohen's d.

Furukawa's method and Kraemer's method to convert Cohen's d into NNT are therefore at odds with each other. This paper aims to empirically examine and compare these two approaches, based on the individual patient data of randomized controlled trials of second generation antipsychotics in the acute phase treatment of patients with schizophrenia.

## Methods

### Database

Individual patient data from 10 trials comparing olanzapine vs placebo (2 comparisons, baseline n = 502) [10], [11], olanzapine vs haloperidol (5 comparisons, baseline n = 2974) [10], [12], [13], [14], [15], and amisulpride vs haloperidol (4 comparisons, baseline n = 1198) [16], [17], [18], [19] in the acute phase treatment of schizophrenia that administered either the BPRS or PANSS were reanalyzed *post hoc*. One trial was a three-armed trial among olanzapine, haloperidol and placebo, and contributed to two comparisons. Important characteristics of the included studies are presented in Table 1.

**Table 1:** Characteristics of the included studies.

Study	Antipsychotic drugs and daily dosage (mg)	Sample size (n)	Mean BPRS at baseline
Beasley et al 1996 [11]	Olanzapine 10Placebo	5050	55.2
Beasley et al 1996 [10]	Olanzapine 10–15Haloperidol 15Placebo	1336968	59.9
Beasley et al 1997 [12]	Olanzapine 10–15Haloperidol 15	17581	59.1
Tollefson et al 1997 [15]	Olanzapine 5–20Haloperidol 5–20	1337659	51.5
Lieberman et al 2003 [14]	Olanzapine 5–20Haloperidol 2–20	131132	46.8
Keefe et al 2006 [13]	Olanzapine 5–20Haloperidol 2–19	15997	48.4
Möller et al 1997 [**18**]	Amisulpride 600–800Haloperidol 15–20	9596	61.7
Puech et al 1998 [19]	Amisulpride 400–1200Haloperidol 16	19464	61.3
Colonna et al 2000 [17]	Amisulpride 200–800Haloperidol 5–20	368118	56.2
Carrière et al 2000 [**16**]	Amisulpride 400–1200Haloperidol 10–30	97105	65.4

BPRS: Brief Psychiatric Rating Scale, CGI-S: Clinical Global Impression Severity Scale, DSM: Diagnostic and Statistical Manual of Mental Disorders, PANSS: Positive and Negative Syndrome Scale.

All studies were randomized and all but one [17] were described as double-blind. All amisulpride studies and one olanzapine study [10] used the original BPRS, and all the other olanzapine studies used PANSS. For the latter studies we calculated the PANSS-derived BPRS scores because PANSS includes all items of the BPRS.

For fixed-dose studies, we selected only those arms with optimum doses of second-generation antipsychotic drugs as reported in dose-finding studies (amisulpride 400–800 mg/day, olanzapine 10–20 mg/day and risperidone 4–6 mg/day) [20]. We therefore excluded 61 participants from Puech et al (1998) [19] who had received a potentially subtherapeutic 100 mg/day of amisulpride, 175 participants from Beasley et al (1997) [12] who received 5 mg/day or 1 mg/day of olanzapine, 65 participants from Beasley et al 1996 [10] who were given 5 mg/day of olanzapine and 52 participants from Beasley et al 1996 [11] who received 1 mg/day of olanzapine.

The mean BPRS total score of the included participants was 54.3 (SD = 10.8) at baseline. There were 2895 men and 1383 women. Their mean age was 36.6 (10.5) years, weight 75.5 (16.4) kg and height 171.6 (9.6) cm.

### Statistical analyses

We first conducted meta-analyses of the BPRS or PANSS total score at 4 weeks for the three comparisons of olanzapine vs haloperidol, amisulpride vs haloperidol and olanzapine vs placebo, using Review Manager software by the Cochrane Collaboration [21]. 4-week was chosen because all the studies reported BPRS at this point in time. Following the strict intention-to-treat principle, missing data were supplemented by the last-observation-carried-forward (LOCF) method even when a participant dropped out before the first post-baseline rating. Unless statistically significant heterogeneity was noted, we obtained the standardized mean difference (Cohen's d) based on the Mantel-Haenszel fixed effect model.

We next calculated the numbers of responders defined as 10% through 90% reduction on the BPRS or PANSS total score at 4 weeks. The percentage reduction was calculated according to the formulae: B% = (B_0_−B_4LOCF_) * 100/(B_0_−18) for BPRS and P% = (P_0_−P_4LOCF_) * 100/(P_0_−30) for PANSS, where B_0_ and P_0_ are BPRS and PANSS scores at baseline and B_4_ and P_4_ are respective scores at 4 weeks, because 18 and 30 are the minimum scores for BPRS and PANSS, respectively, according to the original rating system. We then ran meta-analyses of response rates defined as 10% through 90% reduction for each comparison in terms of risk difference. The pooled NNT was obtained by taking the inverse of this pooled risk difference, because the response rates for a certain cutoff did not differ substantively among the trials included in the meta-analysis, [22].

These actual NNTs were then compared with NNTs converted from Cohen's d according to Kraemer's method and to Furukawa's method using the formulae discussed in the Introduction. The agreement between the actual and the converted was quantified by ANOVA intraclass correlation coefficient (two-way mixed effects, absolute agreement, single measure) by using SPSS Version 17.

## Results

No statistical heterogeneity was observed for any of the meta-analytic summaries. Table 2 tabulates the observed NNTs, NNTs converted from Cohen's d according to Kraemer's method and those according to Furukawa's method for the three comparisons of olanzapine vs haloperidol, amisulpride vs haloperidol and olanzapine vs placebo on BPRS and for the comparison of olanzapine vs haloperidol on PANSS. All but one of the estimated NNTs according to Furukawa's method were included in the 95% confidence intervals of the observed NNTs (35 out of 36, 97%), whereas those calculated by Kraemer's method were within those ranges in 20 out of 36 (56%) instances only. It should also be noted that Kraemer's NNTs were almost always smaller than (i.e. overestimates of) the actual NNTs.

**Table 2:** Agreement between the observed NNTs, those converted from Cohen's d according to Kraemer's method and those according to Furukawa's method for various definitions of response.

Olanzapine vs placebo (BPRS), d = 0.34				
Definition of response	CER	Actual NNT	Kraemer's method	Furukawa's method
10%	0.42	5.9 (3.4 to 20)	5.3	7.4
20%	0.35	7.1 (4.0 to 50)	5.3	7.6
30%	0.26	6.7 (4.0 to 25)	5.3	8.3
40%	0.21	9.1 (4.5 to 100)	5.3	9.1
50%	0.16	11.1 (5.3 to ∞)	5.3	10.4
60%	0.11	16.7 (−∞ to −50, 7.7 to ∞)	5.3	12.9
70%	0.06	25.0 (−∞ to −50, 9.1 to ∞)	5.3	19.1
80%	0.04	−100 (−∞ to −17, 25 to ∞)	5.3	25.5
90%	0.01	−100 (−∞ to −25, 50 to ∞)	5.3	74.1

Olanzapine vs haloperidol (BPRS), d = 0.17				
Definition of response	CER	Actual NNT (95%CI)	Kraemer's method	Furukawa's method
10%	0.64	12.5 (9.1 to 25)	10.5	16.3
20%	0.52	11.1 (7.7 to 16.7)	10.5	14.9
30%	0.40	11.1 (7.7 to 16.7)	10.5	15.0
40%	0.29	12.5 (9.1 to 25)	10.5	16.5
50%	0.19	14.3 (10 to 25)	10.5	20.2
60%	0.11	25.0 (14.3 to 50)	10.5	28.3
70%	0.06	33.3 (20 to 100)	10.5	43.4
80%	0.02	33.3 (25 to 100)	10.5	102.0
90%	0.005	100 (50 to ∞)	10.5	326.0

Olanzapine vs haloperidol (PANSS), d = 0.17				
Definition of response	CER	Actual NNT	Kraemer's method	Furukawa's method
10%	0.61	12.5 (8.3 to 25)	10.5	15.8
20%	0.47	11.1 (7.7 to 20)	10.5	14.8
30%	0.34	11.1 (7.7 to 20)	10.5	15.6
40%	0.23	14.3 (10 to 25)	10.5	18.2
50%	0.15	20.0 (14.3 to 50)	10.5	23.2
60%	0.09	33.3 (20 to 100)	10.5	33.6
70%	0.04	58.8 (28 to 200)	10.5	62.4
80%	0.01	47.6 (31 to 100)	10.5	162.7
90%	0.004	125 (71 to ∞)	10.5	384.5

Amisulpride vs haloperidol (BPRS), d = 0.21				
Definition of response	CER	Actual NNT	Kraemer's method	Furukawa's method
10%	0.78	16.7 (9.1 to 100)	8.5	17.5
20%	0.67	10.0 (6.3 to 20)	8.5	13.9
30%	0.57	9.1 (5.9 to 20)	8.5	12.4
40%	0.49	10.0 (5.9 to 25)	8.5	12.0
50%	0.38	7.7 (5.3 to 14.3)	8.5	12.2
60%	0.26	9.1 (5.9 to 20)	8.5	13.8
70%	0.17	14.3 (8.3 to 50)	8.5	17.1
80%	0.09	25.0 (12.5 to ∞)	8.5	25.6
90%	0.02	33.3 (20 to 100)	8.5	79.3

BPRS: Brief Psychiatric Rating Scale, PANSS: Positive and Negative Syndrome Scale.

The ANOVA ICC of absolute agreement between the actual NNT and those estimated by Kraemer's method was 0.06 (−0.34 to 0.43, p = 0.39) and that for Furukawa's method was 0.33 (−0.01 to 0.62, p = 0.03). When the response is defined at thresholds as high as 80% or 90% reduction, the CER becomes extremely low and the NNT may be considered degenerate with negative numbers and with 95% confidence intervals extending to infinity. We therefore calculated the ANOVA ICC for the ranges from 10% through 70% reduction where we observed relatively constant OR for these different definitions of response [23]. The ANOVA ICC was 0.12 (−0.16 to 0.45, p = 0.22) for Kraemer's method but 0.86 (0.55 to 0.95, p<0.01) for Furukawa's method.

## Discussion

Each meta-analysis comparing olanzapine vs placebo, olanzapine vs haloperidol, and amisulpride vs haloperidol produces a single Cohen's d. This single effect size was converted into NNTs according to Kraemer's method and Furukawa's method, and compared with the actual NNTs using various cutoffs to define response. NNTs from Kraemer's method overlapped with the observed NNT in 56% of the examined instances but the degree of agreement was at a chance level (ANOVA ICC of 0.12, p = 0.22 at best). Those based on Furukawa's method fell within the observed plausible ranges of NNTs in 97% of the instances and the degree of agreement was ANOVA ICC of 0.86 (0.55 to 0.95, p<0.01) for various definitions of response corresponding with 10% through 70% reduction on the rating scale where we expect to observe a non-small number of responders.

The reason for this difference in performance is that the latter method takes into account the fact that, for a given d on a continuous outcome measure, the response rate can vary depending on the cutoff one adopts to define response. This individualized consideration in assessing clinical importance of Cohen's d is extremely important. For example, d of olanzapine over haloperidol in the acute phase treatment of schizophrenia is approximately 0.17. On the other hand, olanzapine causes more significant weight gain than haloperidol, with an NNH estimated to be around 6 (95%CI: 4–11) [24]. A patient who is normo- to underweight now and who does not have any family and other risk factors for obesity may be happy to try olanzapine to achieve a 30% or more decrease in disease severity. For this patient, given an estimate that 40% of the patients would achieve 30% or more reduction on BPRS when given haloperidol (Cf. Table 2), NNT will be calculated to be 15, and he or she may find this NNT small enough in comparison with NNH for weight gain to justify treatment with olanzapine. On the other hand, another patient who is already somewhat overweight and has multiple family history of diabetes mellitus and cardiovascular diseases may like 70% or more decrease in the BPRS before he/she selects olanzapine over haloperidol. However, because the control event rate for 70% reduction could be as low as 6% and the corresponding NNT may be as large as 43, he/she might reason that trying olanzapine may not be worthwhile.

Converting Cohen'd into NNT is also very important when we argue at the population level. For example, Cohen's d of 0.2 is usually regarded as small effect [25]. However, it corresponds with an NNT of 17 for an event that can happen in 2 out of 10 patients when given the control treatment. “Remission” by an antidepressant treatment is an event that happens at this frequency. In Japan, for example, it is estimated that currently around two million people are receiving antidepressant treatment annually. If we can find a new treatment that is better than the current treatment as usual by Cohen's d of 0.2, it can bring about remission in additional 100 thousand or more people that would not have done so on the current treatment. This of course is no trivial number.

One possible drawback of Furukawa's method is that it requires estimation of control event rate in order to predict NNTs accurately. However we argue that this is more of a strength than a weakness of this method, because this is what EBM practitioners normally do when they apply group-level evidence to individuals [26]. In this connection we would like to emphasize that in the original report of a clinical trial it will be more informative not only to report the overall ES but also the control event rates for different definitions of response in a tabular format [27].

Conversely one can argue that the reason why Kraemer's method turned out to be less efficient is because they subtly re-defined NNT for a continuous outcome as the inverse of the difference between the probability that a patient in the treatment has an outcome preferable to one in the control and the probability that a patient in the control has an outcome preferable to one in the treatment. This definition is slightly different from the conventional definition of NNT in EBM [28].

The interpretation of a quantified effect size is inherently difficult and variable [29], [30], and this is precisely the reason why we have to quantify instead of qualifying. Kraemer's method has been used in several recent meta-analyses to quantify the obtained effect size [7], [8], [9]. Furukawa's method has been cited in the Cochrane Handbook as a way to re-express Cohen's d in terms of NNT [31]. Given the present results, a greater precaution is called for in converting the obtained Cohen's d into one single NNT value according to Kraemer's method. After all, how best to apply group evidence to meet individual patients' needs and values is the defining essence of EBM, and NNT is a means to this end, we therefore had better take individual patients' differences, including their expected event rates, into consideration when we present NNTs to them.
